# Evaluation of mycobacteria infection prevalence and optimization of the identification process in North Sardinia, Italy

**DOI:** 10.1128/spectrum.03179-23

**Published:** 2023-12-07

**Authors:** Xiang Chen, Leonardo Antonio Sechi, Paola Molicotti

**Affiliations:** 1 Department of Biomedical Sciences, University of Sassari, Sassari, Italy; 2 Health Care Center, The First Affiliated Hospital of Shantou University Medical College, Shantou, China; 3 SC Microbiologia, AOU Sassari, Sassari, Italy; Institut National de Santé Publique du Québec, Sainte-Anne-de-Bellevue, Québec, Canada

**Keywords:** *Mycobacterium tuberculosis*, non-tuberculous mycobacteria, drug resistance, identification process

## Abstract

*Mycobacterium tuberculosis* (MTB) and non-tuberculous mycobacteria (NTM) are the most common pathogens of mycobacterial infection, and their prevalence varies around the world. This study aimed to estimate the prevalence of mycobacterial infection and drug resistance in North Sardinia, Italy and to optimize the process of mycobacteria identification. From January 2020 to April 2023, samples from 1,836 suspected individuals in North Sardinia were analyzed. Conventional methods (microscopy smear and culture) and molecular methods (polymerase chain reaction, DNA probe, and mass spectrum) were used in MTB and NTM identification. Among 1,836 suspected individuals, 89 were MTB-positive and 44 were NTM-positive. Bronchial aspiration and bronchoalveolar lavage performed well as substitute specimens for sputum in MTB detection. For drug resistance, 71.91% of MTB and 83.33% of NTM were resistant to at least one antibiotic. The average turn-around time of MTB, slowly growing mycobacteria, and rapidly growing mycobacteria was 7.12, 48.87, and 11.27 days, respectively. Overall, our results show that the prevalence of mycobacterial infection in North Sardinia is relatively optimistic. However, the high proportion of drug resistance detected in the isolates is alarming. The implementation of advanced techniques could help optimize the process of mycobacteria identification.

**IMPORTANCE:**

Mycobacterial infection is a major threat to public health worldwide. Accurate identification of infected species and drug resistance detection are critical factors in treatment. We focused on shortening the turn-around time of identifying mycobacteria species and antibiotic resistance tests.

## INTRODUCTION

Mycobacterial infection is still a major threat to public health worldwide. *Mycobacterium tuberculosis* (MTB) is the most common pathogen, causing a highly infectious disease. According to WHO’s Global Tuberculosis Report 2022, the global number of newly diagnosed cases is about 6.4 million annually (1). It is the leading cause of death from a single infectious agent. Approximately, 1.6 million deaths caused by tuberculosis was recorded in 2021 (1). The other common pathogen of mycobacterial infection is non-tuberculous mycobacteria (NTM). NTM refers, in particular, to a group of mycobacteria other than MTB and *Mycobacterium leprae*, comprising more than 190 species and 14 subspecies (2). NTM are ubiquitous in the environment. Most of them are opportunistic pathogens to humans with few exceptions (3). Recent reports of an increase in the prevalence of NTM pulmonary disease (NTM-PD) in developed countries such as America, Japan, and European countries have drawn our attention (4 – 7). In some low-incidence places, the prevalence of NTM-PD is even surpassing tuberculosis (8).

High accuracy and efficiency of MTB and NTM diagnosis are essential for controlling mycobacterial infection. But to date, microscopy smear and culture are still the most widely used diagnostic tests for mycobacteria identification (9). These conventional methods are reliable and economical. However, ensuring the accuracy of microscopy diagnosis requires considerable expertise and culture is time-consuming. Therefore, new techniques must be introduced to this field. Researchers have made great progress in the rapid and accurate diagnosis of mycobacteria through the implementation of molecular methods such as polymerase chain reaction (PCR) and gene sequencing (10 – 12). Besides, matrix-assisted laser desorption ionization–time of flight mass spectrometry (MALDI-TOF MS) also shows its great potential in the clinical microbiology laboratory (13, 14). This technology generates the mass spectral fingerprints that are unique for each bacterium and identifies them by database comparison (15). These allow us to identify bacteria quickly and precisely at the species level. For the therapy of mycobacterial infection, drug resistance is a great challenge. The drug-resistant form of MTB might be responsible for a quarter of the deaths tuberculosis caused (16). Isoniazid resistance caused by mutations in the *katG* gene and rifampicin resistance caused by mutations in the *rpoB* gene are the most common (17). Moreover, a recently found *resR* mutation did not directly lead to resistance but shortened the post-antibiotic effect (18). These mutations could increase treatment failure and recurrence rates. Either MTB or NTM, the emergence of antibiotic resistance leaves us with few therapeutic options (19 – 21). Consequently, timely identification of drug resistance, which could guide clinicians in choosing antibiotics, is needed.

In this study, we identified mycobacterial infection in different types of specimens by conventional methods (microscopy smear and culture). The definite diagnosis of MTB infection was done by real-time PCR. MALDI-TOF MS and DNA probe assay were used to identify NTM species. Meanwhile, we completed the antibiotic susceptibility test (AST) and MTB drug resistance-associated mutation genes detection. Through these analyses, we aimed to estimate the prevalence of mycobacterial infection and drug resistance in North Sardinia of Italy and optimize the mycobacteria identification process.

## MATERIALS AND METHODS

### Sample collection

Samples of suspected mycobacterial infection were collected from the health system of North Sardinia. All samples were sent to the Biosafety Protection Level-3 Mycobacteriology Laboratory of the Microbiology and Virology Department of AOU Sassari to be processed. Sample processing was carried out in the Biosafety Cabinet and strictly followed the procedure of biosafety protection. The main specimen for mycobacterial identification is sputum. Other specimens, such as urine (UR), bronchial aspiration (BRA), pleural effusion (PL), gastric aspiration (GA), and bronchoalveolar lavage (BAL), that were available for identification would be collected if possible. All the subjects were informed that their samples would be used for Mycobacteria identification and antibiotic susceptibility tests.

### Microscopy smear

Smears were prepared from each sample with acid fast staining using the Ziehl Neelsen Kit (Liofichem Diagnostics, Teramo, Italy). Then, they were processed through microscopy to confirm the positivity of acid-fast bacteria.

### Culturing of mycobacteria

After decontamination, all the samples were cultured in both the Lowenstein-Jensen (L-J) solid medium and Mycobacterium Growth Indicator Tube (MGIT) liquid medium. The solid medium was incubated at 37°C in microbiological incubators. The liquid medium was incubated in Bactec MGIT 960 mycobacterial detection system (Becton, Dickinson and Company, NJ, USA).

### Identification of MTB by real-time PCR

DNA extraction of the samples was performed in Microlab Nimbus automated liquid handling platform (Hamilton, Bonaduz, Switzerland) using the Anyplex MTB/NTMe Real-time Detection Kit (Seegene Inc, Seoul, South Korea). Amplification and analysis were performed in the CFX96 Real-time PCR Detection System (Bio-Rad, CA, USA).

### Antibiotic susceptibility test

BD Bactec MGIT 960-SIRE-Kit and BD Bactec MGIT 960-PZA-Kit (Becton, Dickinson and Company, NJ, USA) were used for streptomycin, isoniazid, rifampicin, ethambutol, and pyrazinamide-resistant tests of MTB. Sensititre MIC susceptibility plates (Thermo Scientific, MA, USA) were used for the AST of NTM. The assessment of drug resistance was based on the AST breakpoints released by the Clinical and Laboratory Standards Institute.

### MTB drug resistance-associated mutation genes’ detection

This detection includes mutations associated with isoniazid, rifampicin, fluoroquinolones, and injectable drug resistance. DNA extraction of the samples was performed in Microlab Nimbus automated liquid handling platform (Hamilton, Bonaduz, Switzerland) using the Allplex MTB/MDR/XDRe Detection Kit (Seegene Inc, Seoul, South Korea). Amplification and analysis were performed in the CFX96 Real-time PCR Detection System (Bio-Rad, CA, USA).

### Identification of NTM by DNA probe assay and MALDI-TOF-MS

INNO-LiPA Mycobacteria v2 (Fujirebio Europe N.V., Ghent, Belgium) DNA probe assay was used in NTM species identification. MALDI-TOF-MS was based on the Vitek MALDI-TOF mass spectrometry identification system (Biomérieux, Lyon, France).

### Figure processing and data analysis

Figures were created with Biorender.com and GraphPad Prism (version 9.0, Dotmatics). Data analysis was performed in SPSS Statistics (version 26.0, IBM Corporation, NY, USA). Categorical variables were presented in the form of proportion, and continuous variables were presented in the form of mean and SD. The calculation of turn-around time (TAT) was from the day the samples were accepted to the day pathogens were identified.

## RESULTS

### Study population and mycobacteria identification results

**Fig 1:**
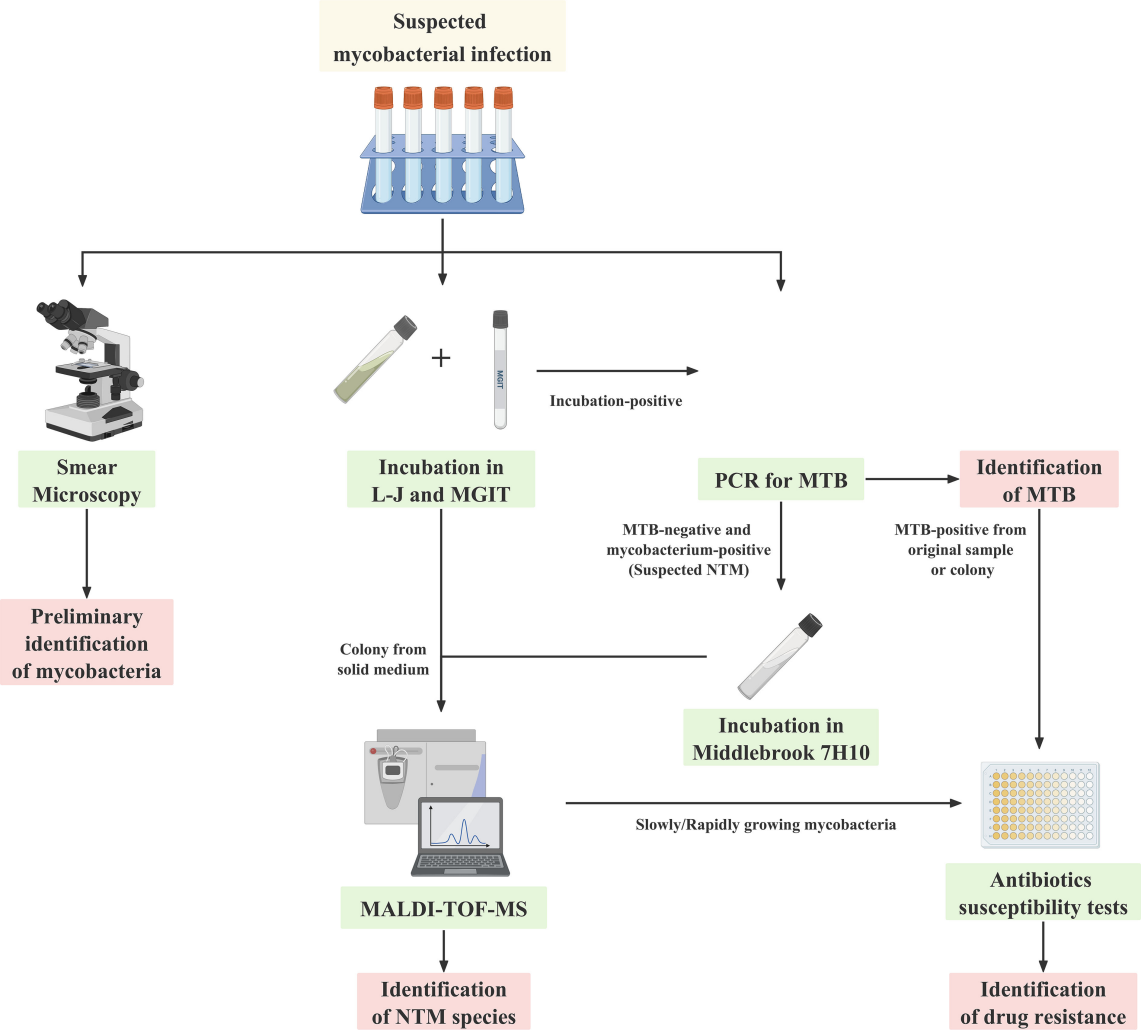
Flowchart of mycobacteria identification.

The sample collection of suspected mycobacterial infections lasted from January 2020 to April 2023. A total of 1,836 individuals including 1,092 males and 744 females were involved in this study. The baseline characteristics of the study population are summarized in Table 1. The flowchart of mycobacteria identification is shown in Fig. 1. Among the 1,836 individuals with suspected mycobacterial infection, 89 were MTB-positive and 42 were NTM-positive. The average age of MTB and NTM infections was 54.96 ± 20.72 and 64.07 ± 16.99 years, respectively. Of the 42 NTM infections identified, there were 29 slowly growing mycobacteria (SGM), 11 rapidly growing mycobacteria (RGM), and 2 unknown species. The average TAT of MTB was 7.12 days, and it was 48.86 days in SGM and 11.27 days in RGM.

**TABLE 1:** Characteristics of the study population

Characteristics		MTB (*n* = 89)	NTM (*n* = 42)	Negative (*n* = 1,705)	Total (*n* = 1,836)
Age		54.96 ± 20.72	64.07 ± 16.99	63.69 ± 16.77	63.30 ± 17.07
Gender	Male	59 (66.29%)	20 (47.62%)	1,013 (59.41%)	1,092 (59.48%)
	Female	30 (33.71%)	22 (52.38%)	692 (40.59%)	744 (40.52%)

### MTB-positive results in different types of specimens

**Fig 2:**
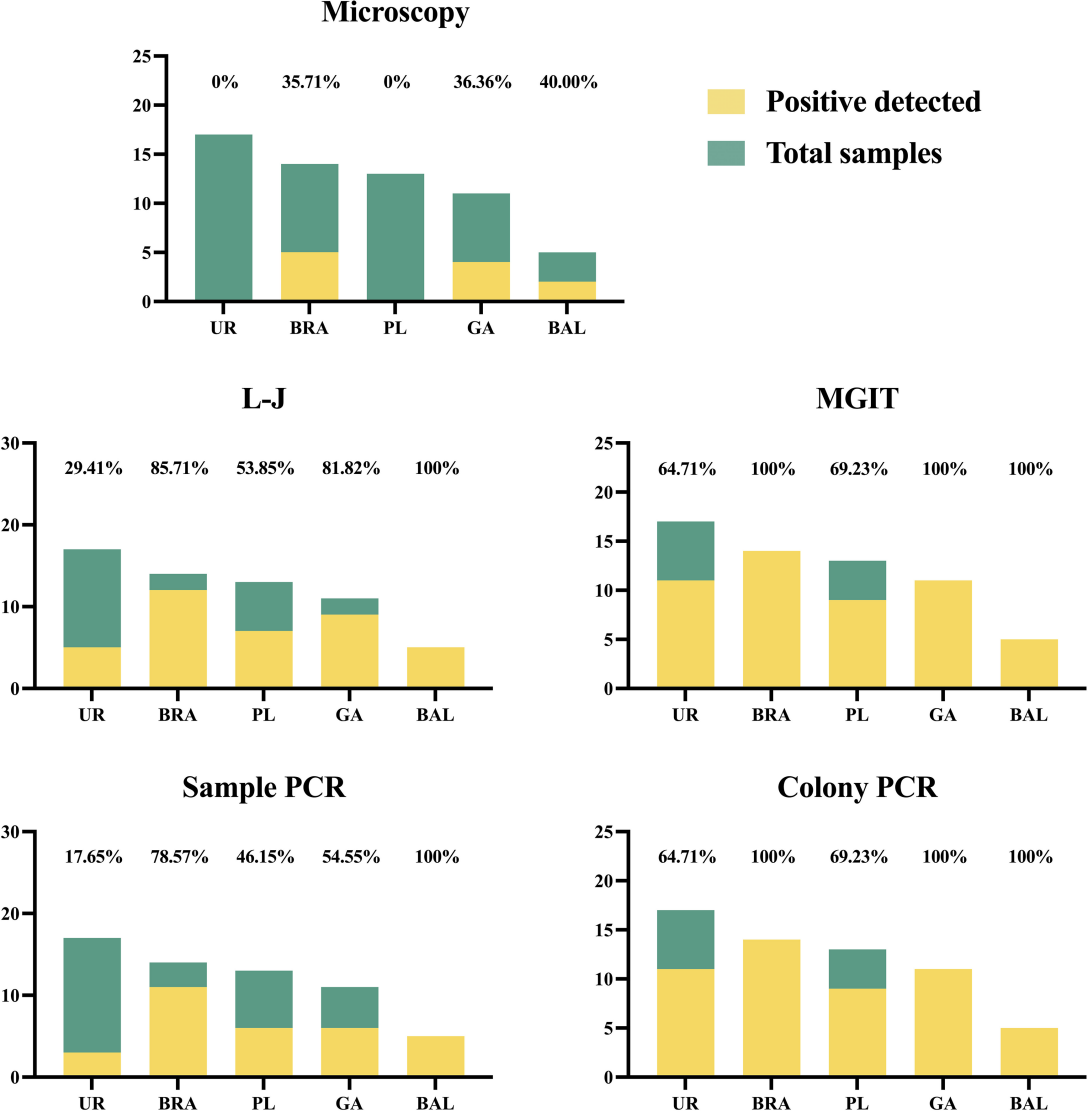
MTB-positive samples detected in different types of specimens.

The positive rates of sputum were 65.17% (58/89) under microscopy, 89.89% (80/89) in L-J culture, and 100% (89/89) in MGIT culture, respectively. They were 82.02% (73/89) in the original samples’ PCR and 100% (89/89) in colony PCR. For other specimens, as Fig. 2 and Table 2 shows, it was unable to identify MTB-positive from UR (0/17) and PL (0/13) in the microscopy smear. The positive rate of BRA, GA, and BAL was 35.71% (5/14), 36.36% (4/11), and 40% (2/5), respectively. For L-J incubation, the positive rate of UR, BRA, PL, GA, and BAL was 29.41% (5/17), 85.71% (12/14), 53.85% (7/13), 81.82% (9/11), and 100% (5/5), respectively. For MGIT incubation, all samples of BRA, GA, and BAL got positive results. The positive rate of UR was 64.71% (11/17) and PL was 69.23% (9/13). In PCR identification, the result of UR, BRA, PL, GA, and BAL using the original sample was 17.65% (3/17), 78.57% (11/14), 46.15% (6/13), 54.55% (6/11), and 100% (5/5). Besides those UR and PL samples not growing in the culture medium, the results of PCR using colonies from incubation were all 100% positive.

**TABLE 2:** MTB-positive detected in different types of specimens

	Microscopy	L-J	MGIT	Sample PCR	Colony PCR
Sputum (*n* = 89)	58	80	89	73	89
UR (*n* = 17)	0	5	11	3	11
BRA (*n* = 14)	5	12	14	11	14
PL (*n* = 13)	0	7	9	6	9
GA (*n* = 11)	4	9	11	6	11
BAL (*n* = 5)	2	5	5	5	5

### Antibiotic susceptibility and mutation genes’ test results of MTB-positive samples

**Fig 3:**
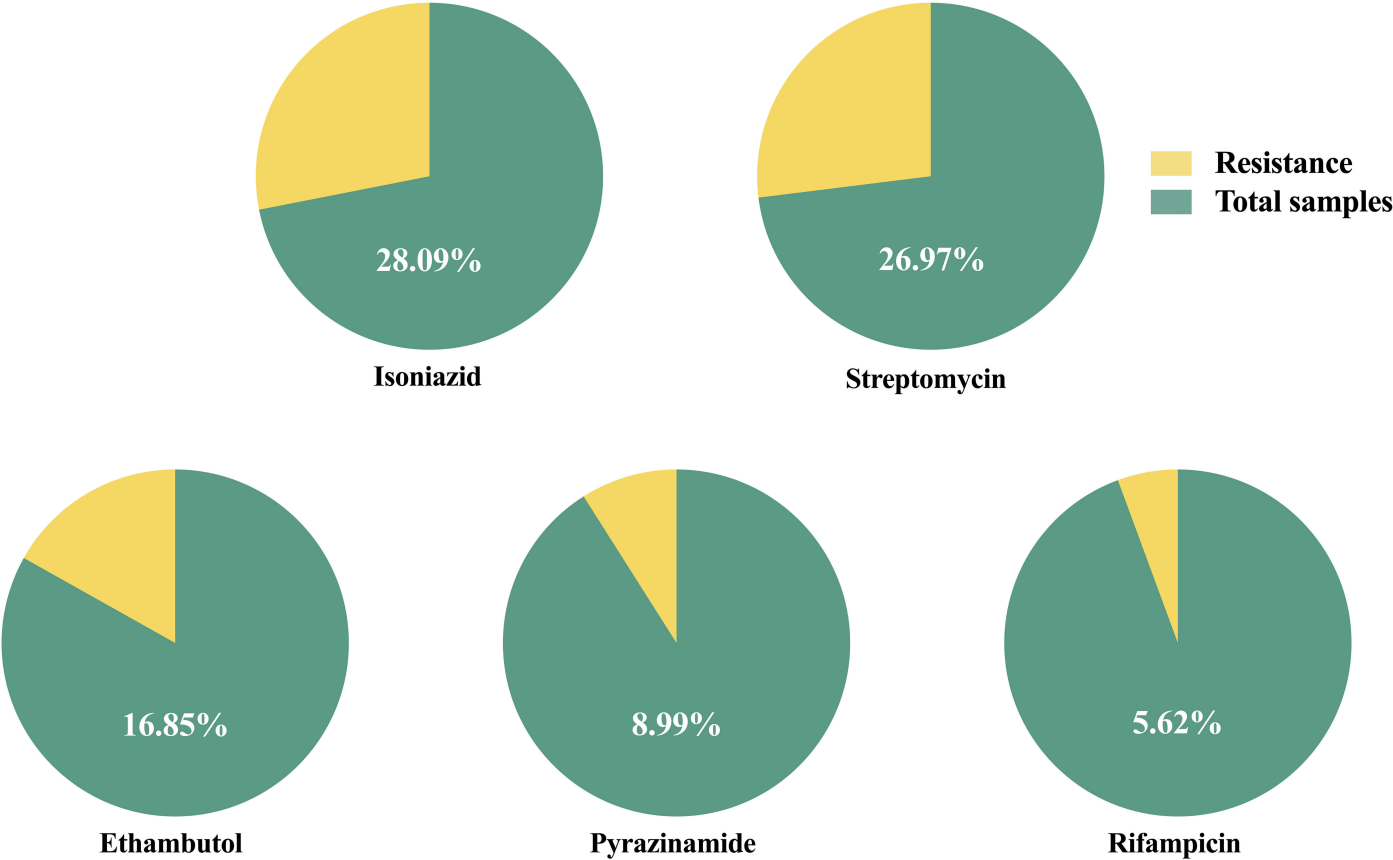
Antibiotic susceptibility test results of MTB-positive samples.

Antibiotic susceptibility tests of five first-line anti-tuberculosis drugs (streptomycin, isoniazid, rifampicin, ethambutol, and pyrazinamide) in MTB treatments were done for all MTB-positive samples. A total of 64 drug-resistant bacteria were detected from 89 MTB-positive individuals. The AST results (Fig. 3) show that the most common type of drug resistance in MTB is isoniazid, which achieves a rate of 28.09% (25/89). Streptomycin comes second with a rate of 26.97% (24/89). The resistance rate of ethambutol and pyrazinamide was 16.85% (15/89) and 8.99% (8/89), respectively, and 5.62% (5/89) were resistant to rifampicin. The detection of mutation genes was only presented in 34 MTB-positive samples from 2022 to 2023. There were two isoniazid resistance and one rifampicin resistance detected. The sensitivity was 20% and 33.33%, respectively. The specificity was 100%. The positive predictive value (PPV) was 100%. The negative predictive value (NPV) was 75% and 93.94%, respectively. No fluoroquinolones and injectable drug resistance were detected.

### Species identification and antibiotic susceptibility test results of NTM

**Fig 4:**
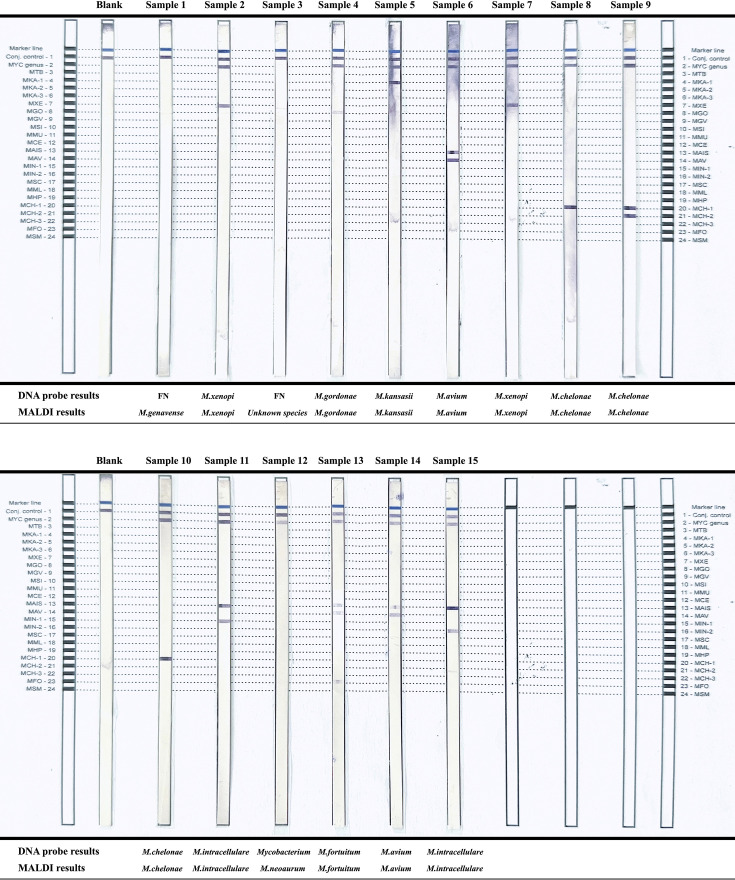
Comparison of DNA probe and MALDI results. Location of probes: 1. FN, false negative; 2. MYC genus, *Mycobacterium*; 3. MTB, *M. tuberculosis* complex; 4. MKA-1, *M. kansasii* (group I); 5. MKA-2, *M. kansasii* (group II); 6. MKA-3, *M. kansasii* (groups III, IV, and V) and *Mycobacterium gastri*; 7. MXE, *M. xenopi*; 8. MGO, *M. gordonae*; 9. MGV, *M. genavense*; 10. MSI, *Mycobacterium simiae*; 11. MMU, *Mycobacterium marinum* and *Mycobacterium ulcerans*; 12. MCE, *Mycobacterium celatum*; 13. MAIS, *M. avium*, *M. intracellulare*, *Mycobacterium scrofulaceum*, and *Mycobacterium malmoense*; 14. MAV, *M. avium*, *Mycobacterium paratuberculosis*, and *Mycobacterium silvaticum*; 15. MIN-1, *M. intracellulare* (sequevars Min-A, -B, -C, and -D); 16. MIN-2, *M. intracellulare* (sequevar Mac-A); 17. MSC, *M. scrofulaceum*; 18. MML, *M. malmoense*; 19. MHP, *Mycobacterium haemophilum*; 20. MCH-1, *M. chelonae* (groups I, II, III, and IV); 21. MCH-2, *M. chelonae* (group III); 22. MCH-3, *M. chelonae* (group I); 23. MFO, *M. fortuitum*; and 24. MSM, *Mycobacterium smegmatis*.

Among the 42 NTM identified by MALDI-TOF MS, there were 29 SGM including nine *Mycobacterium avium*, one *Mycobacterium genavense*, four *Mycobacterium gordonae*, eight *Mycobacterium intracellulare*, one *Mycobacterium kansasii*, one *Mycobacterium lentiflavum*, and five *Mycobacterium xenopi*. There were two unknown species and 11 RGM including three *Mycobacterium abscessus*, four *Mycobacterium chelonae*, three *Mycobacterium fortuitum*, and one *Mycobacterium neoaurum*. As shown in Table 3, only 26.19% (11/42) of the NTM-positive samples could be identified in the microscopy smear. The positive rate of culturing was 78.57% (33/42) in L-J and 100% (42/42) in MGIT, respectively. The comparison of DNA probe assay and MALDI results was done in 15 samples from 2021 to 2022. There were two false negatives in the DNA probe, which were identified as *M. genavense* and unknown species by MALDI. The *M. neoaurum* sample was only identified at the *Mycobacterium* genus level in the DNA probe. Other samples (12/15) achieved consistent results (Fig. 4). For the DNA probe, the sensitivity was 86.67% and specificity was 100%. PPV and NPV were 100% and 33.33%, respectively.

**TABLE 3:** The NTM-positive rate in microscopy and incubation

NTM species	Microscopy	L-J	MGIT
Slowly growing NTM			
*M. avium* (*n* = 9)	3	9	9
*M. genavense* (*n* = 1)	0	0	1
*M. gordonae* (*n* = 4)	0	0	4
*M. intracellulare* (*n* = 8)	1	7	8
*M. kansasii* (*n* = 1)	0	0	1
*M. lentiflavum* (*n* = 1)	0	0	1
*M. xenopi* (*n* = 5)	1	5	5
Rapidly growing NTM			
*M. abscessus* (*n* = 3)	2	3	3
*M. chelonae* (*n* = 4)	3	4	4
*M. fortuitum* (*n* = 3)	1	2	3
*M. neoaurum* (*n* = 1)	0	1	1
Unknown species (*n* = 2)	0	2	2
Total (*n* = 42)	11 (26.19%)	33 (78.57%)	42 (100%)

AST was done for 24 positive samples (16 SGM and 8 RGM) considered to be clinically significant. Among the SGM, three are clarithromycin-resistant, three are ethambutol-resistant, four are isoniazid-resistant, five are moxifloxacin-resistant, three are rifampicin-resistant, four are amikacin-resistant, seven are linezolid-resistant, four are ciprofloxacin-resistant, four are streptomycin-resistant, and two are doxycycline-resistant. Among the RGM, three are ciprofloxacin-resistant, two are moxifloxacin-resistant, one is amikacin-resistant, five are doxycycline-resistant, two are clarithromycin-resistant, two are linezolid-resistant, and three are tobramycin-resistant.

## DISCUSSION

After testing all suspected samples, we had an overview of the prevalence of mycobacterial infection in North Sardinia, Italy. The estimated average incidence rate of tuberculosis was 3.26 cases per 100,000 population per year over the study period. Comparing to the historical statistics of Sardinia in the 1990s (approximately 26 cases per 100,000 population), it was an 87% decrease (22). At the national level, the overall incidence rate was also downward in Italy in the past two decades according to the Tuberculosis surveillance and monitoring reports from ECDC (European Centre for Disease Prevention and Control) (23, 24). For NTM, it was 1.54 cases per 100,000 population per year during the study. Although there is no historical data on Sardinia or Italy to compare, this number was not as bad as described in other European countries (6, 7). Remarkable geographic differences in prevalence were also observed by other researchers. In a study of people aged 65 or older in the United States, the prevalence was over 200 cases per 100,000 persons in some states, while some have data of less than 50 cases (4). The data reflected a higher prevalence in elders than average as well. The mean age of NTM-positive cases (64.07 ± 16.99) in our study was also significantly higher than the MTB-positive cases (54.96 ± 20.72). This implied that the aging population might be one of the reasons for the increasing NTM-PD incidence in developed countries. An immunocompromised state or an underlying disease in elders might increase the chance of infection (25).

In the diagnosis of mycobacterial infection, conventional methods are still irreplaceable. Culture is the current gold standard. Microscopy smear also plays an important role in the microbiology laboratory, although its positive rates could be affected by sample quality, the smear preparation, and the detection technique of the operator (9). The result of the microscopy smear seems to be associated with the MTB-positive rate of sample PCR and TAT. Our results showed that the positive rate of PCR was 98.28% (57/58) in the smear-positive samples but only 51.61% (16/31) in the smear-negative samples. The average TAT in the smear-positive samples was 4.45 days but was 12.26 days in the smear-negative samples. A smear-negative approximates a low number of mycobacteria in the sample. This means the extracted DNA amount might not reach the minimum detection limitation, which leads to suboptimal sensibility in PCR (26, 27). Different types of specimens could also have an impact on mycobacteria identification. As our results showed, urine, PL, and GA might not be ideal specimens but BRA and BAL performed well in identifying MTB. We would recommend BRA or BAL as a substitute for timely identification if sputum samples cannot be obtained from suspected cases in the first place.

Multiple drug resistance (MDR) is the biggest problem we face in treating mycobacterial infections. MDR would significantly increase the risk of treatment failure, relapse, or acquisition of resistance to other drugs (24). The definition of multidrug-resistant tuberculosis (MDR-TB) is MTB not responding to at least isoniazid and rifampicin. Resistance to these two plus any fluoroquinolone and at least one of three injectable second-line drugs could be defined as extensively drug-resistant tuberculosis (28). Stats from ECDC showed that the rifampicin resistance incidence rate is 3.90% in Italy (24). The 5.62% resistance we detected was higher than the average. Rifampicin exerts its bactericidal effect by inhibiting nucleic acid synthesis. Resistance occurred with mutations in the *rpoB* gene encoding the RNA polymerase β-subunit (29, 30). Isoniazid (28.09%) was the most common type of resistance in our study. Although there was no official record available, this was an alarming percentage even compared to the incidence rate in previously treated MTB patients (27.2%) (31, 32). Most isoniazid resistance was caused by mutations at the codon 315 (serine to threonine) of the *katG* gene (33). Mutations in the *inhA* and *ahpC* promoters usually result in low-level resistance. But it would become a high-level resistance when they appeared with *katG* mutation (34). Moreover, of the 64 drug-resistant MTB-positive individuals we identified, 10 of them were resistant to two of the first-line therapy antibiotics, and eight were resistant to three. Even one of them was resistant to all of the five first-line drugs. For the NTM, the most common resistances detected in our study are linezolid and fluoroquinolone (ciprofloxacin and moxifloxacin). Linezolid resistance was usually induced by 23S rRNA gene mutation, while fluoroquinolone resistance was induced by DNA gyrase or topoisomerase mutation (35, 36). The AST results of NTM are grim too. Over 80% (20/24) of them are resistant to at least one antibiotic. Hence, a detection kit that covers a qualitative test for MTB and drug-resistant genes was introduced, intending to shorten the time of identifying MDR-TB. The kit successfully identified all the MTB-positive samples. However, the consistency of the mutation gene detection and AST results is not good enough. It only identified the isoniazid resistance mutation gene in two samples, while the results from AST were 10. For the rifampicin-resistance gene, it was only able to identify one of the three resistance samples. Among the rifampicin-resistant samples, mutation genes could not be detected, both of them are smear-negative. Of the eight false-negative isoniazid-resistant samples, six are smear-negative. A similar result was observed in another study, and the sensibility could be improved while using cultured samples (37). This might also relate to the low DNA amount in smear-negative samples as we mentioned above. More smear-positive samples are needed to fully evaluate the kit’s performance in identifying the MDR. On the other hand, a modified method such as droplet digital PCR and multiplex allele-specific PCR could also improve the sensibility of mutation gene detection (38 – 40).

In NTM identification, most isolates in our study are *M. avium* and *M. intracellulare*. Both belong to *Mycobacterium avium* complex, the most common NTM that causes pulmonary disease (41). The infection of these two pathogens might have similar symptoms but the severity is different. *M. intracellulare* is more virulent than *M. avium* (42, 43). In accordance with the authoritative organization’s official guidelines, the treatment of NTM infection differs by its species (44). But there are general antibiotics such as clarithromycin, amikacin, and linezolid for common NTM infections. As one of the steps in optimizing the identification process, a real-time PCR kit that covered both MTB and mycobacteria detection was applied to identify MTB and NTM simultaneously. The result of a negative in MTB with a positive in *Mycobacterium* refers to an NTM, and a result with *Mycobacterium* Ct-value smaller than MTB could be judged as MTB and NTM co-infection. Then, measures of NTM therapy could be taken in time according to the patient’s condition. The need for rapid identification at the species level is also underscored in the guideline (44). Compared to classical tests based on biochemistry, the implementation of MALDI-TOF MS and DNA probe assay apparently improves the efficiency and accuracy of NTM species identification. In our study, most of the NTM samples achieved the same identification result in these two assays. However, there were still two false negatives in the DNA probe assay, which might have been caused by failure in its complicated amplification and hybridization procedure. Besides, the commercial DNA probe assay kits have a fixed *Mycobacterium* species identification list. It only consists of the most common NTM. Other infrequent species such as *M. neoaurum* could only be identified at the genus level. So, from our perspective, MALDI-TOF MS is superior to DNA probe assay due to its less pre-analysis preparation and upgradable database. However, MALDI-TOF MS is still a culture-based test. Previous studies found that subcultures from solid mediums performed better in species identification (14, 45). There are studies reporting poor identification rates of NTM when using a liquid medium (46 – 48). This made a solid culture medium become our first choice. But some of the NTM like *M. gordonae* in our study have no growth in L-J. Therefore, for NTM identification samples, we chose to incubate in a Middlebrook 7H10 agar at the same time to ensure efficiency. What is more, researchers are exploring new molecular and bioinformatic methods for non-culture-based NTM species identification (49 – 51). The clinical application of these techniques could further reduce the TAT of NTM identification.

In conclusion, our results demonstrated that the prevalence of mycobacterial infection in North Sardinia is relatively optimistic, but the situation of drug resistance is not. The process of mycobacteria identification could be optimized by more options in specimen types, real-time PCR implemented in distinguishing MTB and NTM, MALDI-TOF MS applied in NTM species identification, and modification in the workflow. However, the attempt to detect drug-resistant mutation genes was not successful. We believe the TAT of mycobacterial infection diagnosis and drug-resistance detection could be further shortened in the future with the application of more advanced techniques.
